# Supplementary material for: Evaluating Overall Performance in High-Level Dressage Horse–Rider Combinations by Comparing Measurements from Inertial Sensors with General Impression Scores Awarded by Judges
Source: Animals (Basel). 2023 Aug 2;13(15):2496. doi: 10.3390/ani13152496 (PMC10417551; doi:10.3390/ani13152496)
Supplement: Supplementary file 1 [file animals-13-02496-s001.zip › animals-2502231-supplementary.pdf]

Supplementary File S1

**Test for 2020 Florida Study**

Your input will be used; 1) in a research study to compare the judge's scores and comments with gait characteristics measured by sensors attached to the horse's body, and 2) to assist a project supported by FEI to provide evidence to update the classification system for para dressage. We will compare the rider collective scores with sensors attached to the rider and the horse.

| Scale of marks: | |
|---|---|
| 10. Excellent | 5. Sufficient |
| 9. Very Good | 4. Insufficient |
| 8. Good | 3. Fairly Bad |
| 7. Fairly Good | 2. Bad |
| 6. Satisfactory | 1. Very Bad |

- Focus on the quality of the gaits/movements rather than accuracy. A rider score is included at the end of the sheet.
- Judge all gaits and movements at Grand Prix level.
- Comments/observations may be written or spoken if you wish to make any. If spoken, please upload/return an audio file with your score sheet. Comments are not essential but would be nice to have.
- If it is not possible to mark a gait or movement for any reason (for example, the camera has been zoomed in too much), leave a blank score.
- Not all horse/rider combinations performed all the gaits and movements in the test, so the video and test sheet have been edited to include what was performed.
- Breaks in the score sheet indicate breaks in the video.
- For some horses, additional transitions performed on straight lines are shown separately at the end of the video.
- Half marks may be used.

| Approximate location | Gait/Movement | Score (1-10) | Comments/Observations |
|---|---|---|---|
| F to M | Collected trot (on left rein) | | |
| H | Transition to extended trot | | |
| H to K | Extended trot | | |
| K | Transition to collected trot | | |
| A | 20 m circle left | | |
| A | 10 m circle left | | |
| F to R | Shoulder in left | | |
| H to V | Travers left | | |
| F to S | Half pass left | | |
| M to F | Collected trot (right rein) | | |
| A | 20 m circle right | | |
| A | 10 m circle right | | |

| | | | |
|---|---|---|---|
| K to S | Shoulder in right | | |
| M to P | Travers right | | |
| K | Transition to extended trot | | |
| K to H | Extended trot | | |
| H | Transition to collected trot | | |
| M to V | Half pass right | | |
| F to E | Half pass left | | |
| E to M | Half pass right | | |
| | BREAK | | |
| F to X | Extended walk | | |
| X | Transition to collected walk | | |
| X to H | Collected walk | | |
| M to X | Collected walk | | |
| X | Transition to extended walk | | |
| X to K | Extended walk | | |
| F to D | Collected walk | | |
| | Transition to piaffe | | |
| D | Piaffe | | |
| | Transition to passage | | |
| K to E | Passage | | |
| | Transition to piaffe | | |
| X | Piaffe | | |
| | Transition to passage | | |
| B to M | Passage | | |
| | Transition to piaffe | | |
| G | Piaffe | | |
| | Transition to collected walk | | |
| | BREAK | | |
| F to M | Collected canter left lead | | |
| C | 20 m circle left | | |
| C | 10 m circle left | | |
| H to P | Half pass left | | |
| A | Counter canter 20 m circle right | | |
| A | Counter canter 10 m circle right | | |
| Near A | Flying change | | |
| K | Transition to extended canter | | |

| | | | |
|---|---|---|---|
| K to H | Extended canter right lead | | |
| H | Transition to collected canter | | |
| M to F | Collected canter right lead | | |
| A | 20 m circle right | | |
| A | 10 m circle right | | |
| K to R | Half pass right | | |
| C | Counter canter 20 m circle left | | |
| C | Counter canter 10 m circle left | | |
| Near C | Flying change | | |
| H | Transition to extended canter | | |
| H to K | Extended canter left lead | | |
| K | Transition to collected canter | | |
| F to E | Change rein with pirouette over centerline (judge pirouette only) | | |
| Near E | Flying change | | |
| E to M | Change rein with pirouette over centerline (judge pirouette only) | | |
| Near M | Flying change | | |
| HXF | Four tempi changes | | |
| KXM | Three tempi changes | | |
| HXF | Two tempi changes | | |
| KXM | One tempi changes | | |
| H to B | Half pass left and | | |
| Near B | Flying change | | |
| B to K | Half pass right | | |
| Near K | Flying change | | |
| A | Transition to collected walk | | |
| | BREAK | | |

**Collective Marks (rider)**

| | **Criteria** | **Score (1-10)** | **Comments/Observations** |
|---|---|---|---|
| | Rider position | | |
| | Rider harmony with the horse | | |
| | Rider correctness and effects of the aids | | |
